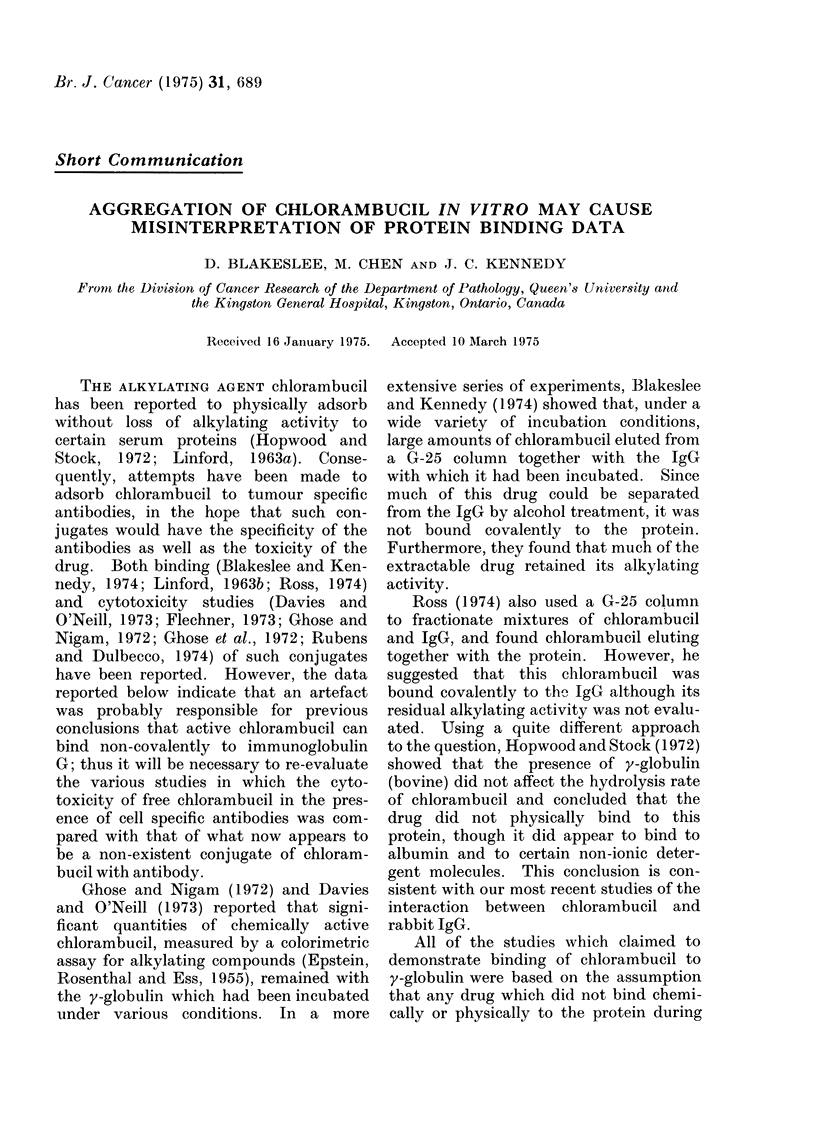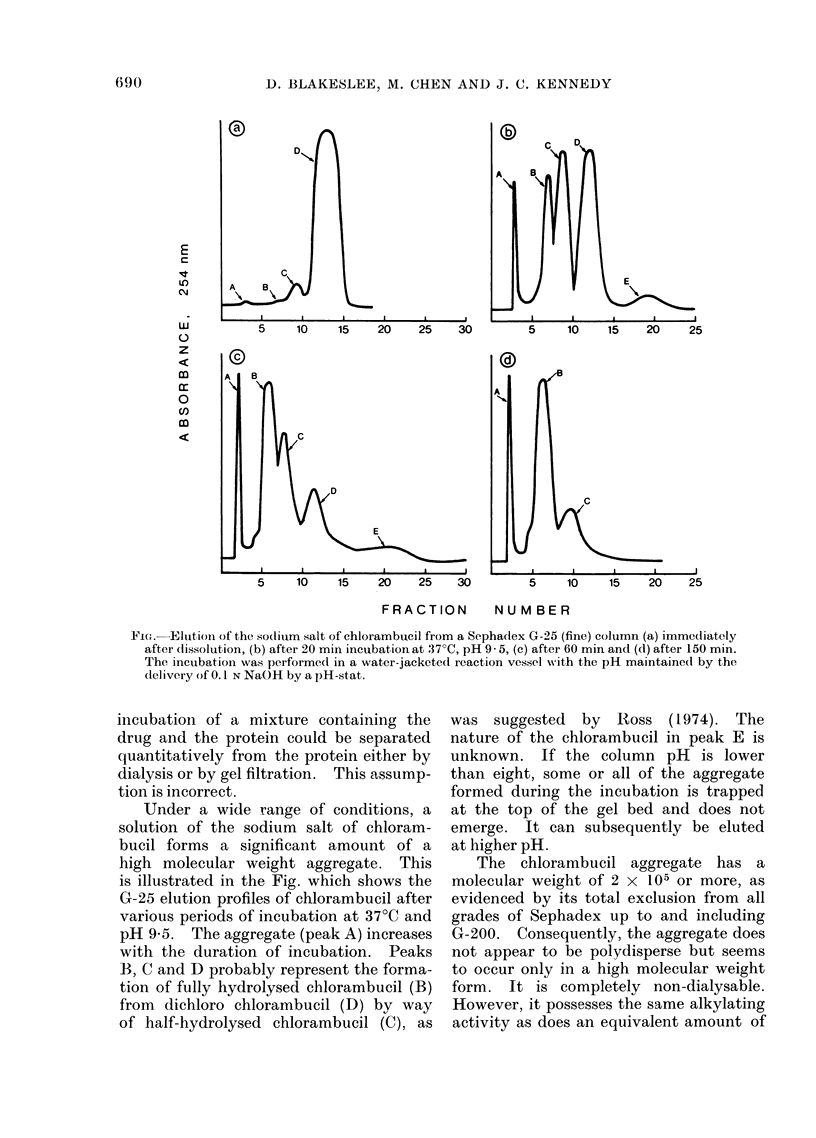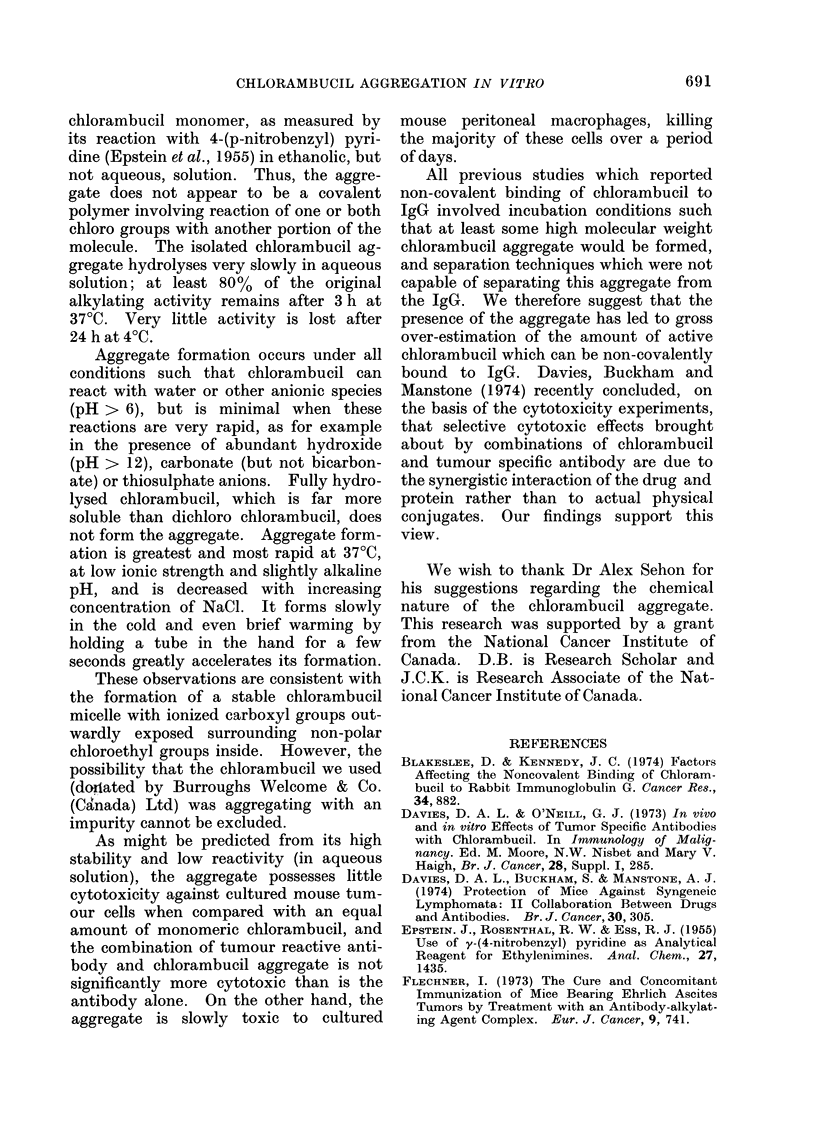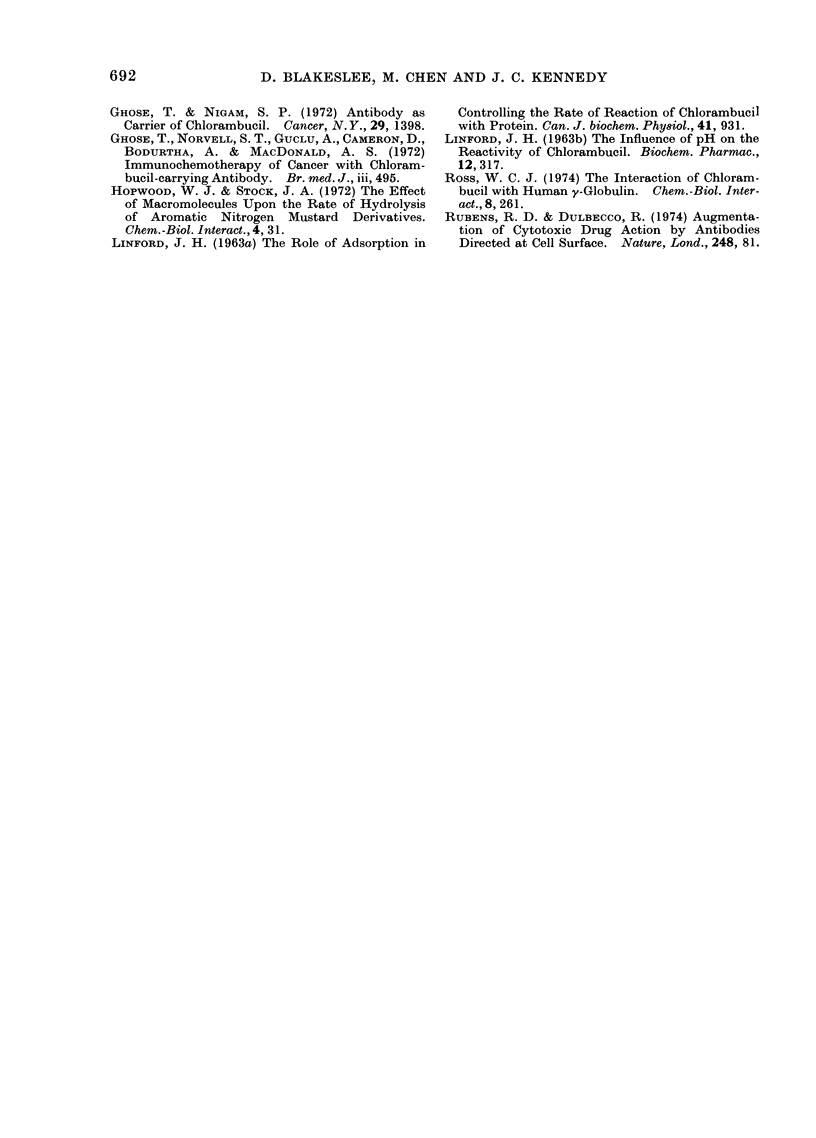# Aggregation of chlorambucil in vitro may cause misinterpretation of protein binding data.

**DOI:** 10.1038/bjc.1975.117

**Published:** 1975-06

**Authors:** D. Blakeslee, M. Chen, J. C. Kennedy


Br. J. Cancer (1975) 31, 689

***Short Communication***

### AGGREGATION OF CHLORAMBUCIL *IN VITRO* MAY CAUSE MISINTERPRETATION OF PROTEIN BINDING DATA

D. BLAKESLEE, M. CHEN AND J. C. KENNEDY

*From the Division of Cancer Research of the Department of Pathology, Queen's University and the Kingston General Hospital, Kingston, Ontario, Canada*

Received 16 January 1975. Accepted 10 March 1975

THE ALKYLATING AGENT chlorambucil has been reported to physically adsorb without loss of alkylating activity to certain serum proteins (Hopwood and Stock, 1972; Linford, 1963*a*). Consequently, attempts have been made to adsorb chlorambucil to tumour specific antibodies, in the hope that such conjugates would have the specificity of the antibodies as well as the toxicity of the drug. Both binding (Blakeslee and Kennedy, 1974; Linford, 1963*b*; Ross, 1974) and cytotoxicity studies (Davies and O'Neill, 1973; Flechner, 1973; Ghose and Nigam, 1972; Ghose *et al.*, 1972; Rubens and Dulbecco, 1974) of such conjugates have been reported. However, the data reported below indicate that an artefact was probably responsible for previous conclusions that active chlorambucil can bind non-covalently to immunoglobulin G; thus it will be necessary to re-evaluate the various studies in which the cytotoxicity of free chlorambucil in the presence of cell specific antibodies was compared with that of what now appears to be a non-existent conjugate of chlorambucil with antibody.

Ghose and Nigam (1972) and Davies and O'Neill (1973) reported that significant quantities of chemically active chlorambucil, measured by a colorimetric assay for alkylating compounds (Epstein, Rosenthal and Ess, 1955), remained with the $\gamma$-globulin which had been incubated under various conditions. In a more extensive series of experiments, Blakeslee and Kennedy (1974) showed that, under a wide variety of incubation conditions, large amounts of chlorambucil eluted from a G-25 column together with the IgG with which it had been incubated. Since much of this drug could be separated from the IgG by alcohol treatment, it was not bound covalently to the protein. Furthermore, they found that much of the extractable drug retained its alkylating activity.

Ross (1974) also used a G-25 column to fractionate mixtures of chlorambucil and IgG, and found chlorambucil eluting together with the protein. However, he suggested that this chlorambucil was bound covalently to the IgG although its residual alkylating activity was not evaluated. Using a quite different approach to the question, Hopwood and Stock (1972) showed that the presence of $\gamma$-globulin (bovine) did not affect the hydrolysis rate of chlorambucil and concluded that the drug did not physically bind to this protein, though it did appear to bind to albumin and to certain non-ionic detergent molecules. This conclusion is consistent with our most recent studies of the interaction between chlorambucil and rabbit IgG.

All of the studies which claimed to demonstrate binding of chlorambucil to $\gamma$-globulin were based on the assumption that any drug which did not bind chemically or physically to the protein during

FIG.—Elution of the sodium salt of chlorambucil from a Sephadex G-25 (fine) column (a) immediately after dissolution, (b) after 20 min incubation at 37°C, pH 9·5, (c) after 60 min and (d) after 150 min. The incubation was performed in a water-jacketed reaction vessel with the pH maintained by the delivery of 0.1 N NaOH by a pH-stat.

incubation of a mixture containing the drug and the protein could be separated quantitatively from the protein either by dialysis or by gel filtration. This assumption is incorrect.

Under a wide range of conditions, a solution of the sodium salt of chlorambucil forms a significant amount of a high molecular weight aggregate. This is illustrated in the Fig. which shows the G-25 elution profiles of chlorambucil after various periods of incubation at 37°C and pH 9·5. The aggregate (peak A) increases with the duration of incubation. Peaks B, C and D probably represent the formation of fully hydrolysed chlorambucil (B) from dichloro chlorambucil (D) by way of half-hydrolysed chlorambucil (C), as was suggested by Ross (1974). The nature of the chlorambucil in peak E is unknown. If the column pH is lower than eight, some or all of the aggregate formed during the incubation is trapped at the top of the gel bed and does not emerge. It can subsequently be eluted at higher pH.

The chlorambucil aggregate has a molecular weight of $2 \times 10^5$ or more, as evidenced by its total exclusion from all grades of Sephadex up to and including G-200. Consequently, the aggregate does not appear to be polydisperse but seems to occur only in a high molecular weight form. It is completely non-dialysable. However, it possesses the same alkylating activity as does an equivalent amount of chlorambucil monomer, as measured by its reaction with 4-(p-nitrobenzyl) pyridine (Epstein *et al.*, 1955) in ethanolic, but not aqueous, solution. Thus, the aggregate does not appear to be a covalent polymer involving reaction of one or both chloro groups with another portion of the molecule. The isolated chlorambucil aggregate hydrolyses very slowly in aqueous solution; at least 80% of the original alkylating activity remains after 3 h at 37°C. Very little activity is lost after 24 h at 4°C.

Aggregate formation occurs under all conditions such that chlorambucil can react with water or other anionic species (pH > 6), but is minimal when these reactions are very rapid, as for example in the presence of abundant hydroxide (pH > 12), carbonate (but not bicarbonate) or thiosulphate anions. Fully hydrolysed chlorambucil, which is far more soluble than dichloro chlorambucil, does not form the aggregate. Aggregate formation is greatest and most rapid at 37°C, at low ionic strength and slightly alkaline pH, and is decreased with increasing concentration of NaCl. It forms slowly in the cold and even brief warming by holding a tube in the hand for a few seconds greatly accelerates its formation.

These observations are consistent with the formation of a stable chlorambucil micelle with ionized carboxyl groups outwardly exposed surrounding non-polar chloroethyl groups inside. However, the possibility that the chlorambucil we used (donated by Burroughs Welcome & Co. (Canada) Ltd) was aggregating with an impurity cannot be excluded.

As might be predicted from its high stability and low reactivity (in aqueous solution), the aggregate possesses little cytotoxicity against cultured mouse tumour cells when compared with an equal amount of monomeric chlorambucil, and the combination of tumour reactive antibody and chlorambucil aggregate is not significantly more cytotoxic than is the antibody alone. On the other hand, the aggregate is slowly toxic to cultured mouse peritoneal macrophages, killing the majority of these cells over a period of days.

All previous studies which reported non-covalent binding of chlorambucil to IgG involved incubation conditions such that at least some high molecular weight chlorambucil aggregate would be formed, and separation techniques which were not capable of separating this aggregate from the IgG. We therefore suggest that the presence of the aggregate has led to gross over-estimation of the amount of active chlorambucil which can be non-covalently bound to IgG. Davies, Buckham and Manstone (1974) recently concluded, on the basis of the cytotoxicity experiments, that selective cytotoxic effects brought about by combinations of chlorambucil and tumour specific antibody are due to the synergistic interaction of the drug and protein rather than to actual physical conjugates. Our findings support this view.

We wish to thank Dr Alex Sehon for his suggestions regarding the chemical nature of the chlorambucil aggregate. This research was supported by a grant from the National Cancer Institute of Canada. D.B. is Research Scholar and J.C.K. is Research Associate of the National Cancer Institute of Canada.

### REFERENCES

BLAKESLEE, D. & KENNEDY, J. C. (1974) Factors Affecting the Noncovalent Binding of Chlorambucil to Rabbit Immunoglobulin G. *Cancer Res.*, **34**, 882.

DAVIES, D. A. L. & O'NEILL, G. J. (1973) *In vivo* and *in vitro* Effects of Tumor Specific Antibodies with Chlorambucil. In *Immunology of Malignancy*. Ed. M. Moore, N.W. Nisbet and Mary V. Haigh, *Br. J. Cancer*, **28**, Suppl. I, 285.

DAVIES, D. A. L., BUCKHAM, S. & MANSTONE, A. J. (1974) Protection of Mice Against Syngeneic Lymphomata: II Collaboration Between Drugs and Antibodies. *Br. J. Cancer*, **30**, 305.

EPSTEIN. J., ROSENTHAL, R. W. & ESS, R. J. (1955) Use of γ-(4-nitrobenzyl) pyridine as Analytical Reagent for Ethylenimines. *Anal. Chem.*, **27**, 1435.

FLECHNER, I. (1973) The Cure and Concomitant Immunization of Mice Bearing Ehrlich Ascites Tumors by Treatment with an Antibody-alkylating Agent Complex. *Eur. J. Cancer*, **9**, 741.

GHOSE, T. & NIGAM, S. P. (1972) Antibody as Carrier of Chlorambucil. *Cancer, N.Y.*, **29**, 1398.

GHOSE, T., NORVELL, S. T., GUCLU, A., CAMERON, D., BODURTHA, A. & MACDONALD, A. S. (1972) Immunochemotherapy of Cancer with Chlorambucil-carrying Antibody. *Br. med. J.*, iii, 495.

HOPWOOD, W. J. & STOCK, J. A. (1972) The Effect of Macromolecules Upon the Rate of Hydrolysis of Aromatic Nitrogen Mustard Derivatives. *Chem.-Biol. Interact.*, **4**, 31.

LINFORD, J. H. (1963*a*) The Role of Adsorption in Controlling the Rate of Reaction of Chlorambucil with Protein. *Can. J. biochem. Physiol.*, **41**, 931.

LINFORD, J. H. (1963b) The Influence of pH on the Reactivity of Chlorambucil. *Biochem. Pharmac.*, **12**, 317.

ROSS, W. C. J. (1974) The Interaction of Chlorambucil with Human $\gamma$-Globulin. *Chem.-Biol. Interact.*, **8**, 261.

RUBENS, R. D. & DULBECCO, R. (1974) Augmentation of Cytotoxic Drug Action by Antibodies Directed at Cell Surface. *Nature, Lond.*, **248**, 81.